# An attempt to simultaneously quantify the polysaccharide, total lipid, protein and pigment in single *Cyclotella cryptica* cell by Raman spectroscopy

**DOI:** 10.1186/s13068-023-02314-2

**Published:** 2023-04-08

**Authors:** Xiufen Wang, Yuehui He, Yuanyuan Zhou, Baohua Zhu, Jian Xu, Kehou Pan, Yun Li

**Affiliations:** 1 The Key Laboratory of Mariculture (Ministry of Education), Ocean University of China, Qingdao, 266003 Shandong China; 2 Single-Cell Center, CAS Key Laboratory of Biofuels, Shandong Key Laboratory of Energy Genetics, Qingdao Institute of Bioenergy and Bioprocess Technology, Chinese Academy of Sciences, Qingdao, China; 3 Shandong Energy Institute, Qingdao, China; 4 Qingdao New Energy Shandong Laboratory, Qingdao, China; 5 Function Laboratory for Marine Fisheries Science and Food Production Processes, Qingdao National Laboratory for Marine Science and Technology, Qingdao, China

**Keywords:** Single-cell Raman spectra (SCRS), *Cyclotella cryptica* (*C. cryptica*), Intracellular energy-dense macromolecules, Partial Least Square Regression (PLSR), Intra-ramanome Correlation Analysis (IRCA)

## Abstract

**Background:**

At present, the conventional methods for determining photosynthetic products of microalgae are usually based on a large number of cell mass to reach the measurement baseline, and the result can only reveal the average state at the population level, which is not feasible for large-scale and rapid screening of specific phenotypes from a large number of potential microalgae mutants. In recent years, single-cell Raman spectra (SCRS) has been proved to be able to rapidly and simultaneously quantify the biochemical components of microalgae. However, this method has not been reported to analyze the biochemical components of *Cyclotella cryptica* (*C. cryptica*)*.* Thus, SCRS was first attempt to determine these four biochemical components in this diatom.

**Results:**

The method based on SCRS was established to simultaneously quantify the contents of polysaccharide, total lipids, protein and Chl-a in *C. cryptica*, with thirteen Raman bands were found to be the main marker bands for the diatom components. Moreover, Partial Least Square Regression (PLSR) models based on full spectrum can reliably predict these four cellular components, with Pearson correlation coefficient for these components reached 0.949, 0.904, 0.801 and 0.917, respectively. Finally, based on SCRS data of one isogenic sample, the pairwise correlation and dynamic transformation process of these components can be analyzed by Intra-ramanome Correlation Analysis (IRCA), and the results showed silicon starvation could promote the carbon in *C. cryptica* cells to flow from protein and pigment metabolism to polysaccharide and lipid metabolism.

**Conclusions:**

First, method for the simultaneous quantification of the polysaccharide, total lipid, protein and pigment in single *C. cryptica* cell are established. Second, the instant interconversion of intracellular components was constructed through IRCA, which is based on data set of one isogenic population and more precision and timeliness. Finally, total results indicated that silicon deficiency could promote the carbon in *C. cryptica* cells to flow from protein and pigment metabolism to polysaccharide and lipid metabolism.

## Background

Excessive anthropogenic CO_2_ emissions are considered to be the main cause of global warming, which has become a significant environmental issue [1, 2]. Photosynthetic organisms have great potential to fix CO_2_ through biotransformation in vivo [3, 4]. Among photosynthetic organisms, microalgae, with the characteristics of wide distribution, fast growth than higher plants, easy to cultivate and high photosynthetic efficiency, have been considered as one of the most environmentally friendly, safe and sustainable candidates to fix CO_2_ [3, 5–8]. In the meanwhile, polysaccharides, lipids, proteins and pigments are major four intracellular energy-dense macromolecules produced by microalgae cells by fixing CO_2_ through photosynthesis [9–15]. However, the conventional methods for the determination of these high energy substances within microalgae cells have some shortcomings: (1) the understanding of cell phenotype almost all comes from the analysis at the population level, which obscures the richness of cell-to-cell variation; (2) a large number of cultured cells are required to reach the baseline of the measurement; (3) Generally speaking, the determination procedure of conventional methods is complex and time-consuming; (4) specific organic reagents need to be ordered, etc. Briefly, these approaches followed by tedious and time-consuming determination process, which was not feasible for mass screening of a particular phenotype from enormous number of potential mutants [16].

To tackle these challenges, researchers have developed Raman spectroscopy. This approach represents the collective Raman spectra of molecules in one cell and provide an intrinsic chemical profile of the cell in a label-free and non-destructive manner [17–19]. For microalgal cell, the Raman spectroscopy has been used to detect their “product spectrum”. For example, the resonance Raman spectroscopy was used to probing the carotenoid content of intact *Cyclotella meneghiniana* cell [20], and the lipid composition of living *Thalassiosira pseudonana* cell was evaluated by Raman spectroscopy [16]. The single-cell Raman spectroscopy (SCRS) also was successfully applied to the label-free and simultaneous quantitative analysis of starch, protein and triacylglycerol in individual cell of *Chlamydomonas reinhardtii* [21]. Therefore, this method of simultaneously measuring the cellular content of target molecular with high throughput and low cost is of great necessary and valuable to development of microalgae strain and synthesis mechanism research [21].

*Cyclotella cryptica* (*C. cryptica*) was considered to have great economic development potential due to their rapid growth, facultative heterotrophic ability, rich in high value-added products, such as polyunsaturated fatty acids and fucoxanthin [22]. It was also found that the porous hierarchical frustule of *C. cryptica* had good hemostatic potentiality [23]. However, the determination of biochemical components of this diatom was mainly based on conventional methods.

The purpose of this study was to establish a simple, noninvasive and rapid method for the determination of multiple biochemical components of *C. cryptica* based on SCRS. Thus, the changes of biochemical phenotypes in *C. cryptica* cells under Si+ (silicon-replete F/2 medium, control group), Si− (silica starvation F/2 medium) and Si−+ (silica starvation F/2 medium followed by silicon-replete F/2 medium) conditions were systematically characterized at the population level based on a variety of conventional method. Meanwhile, SCRS of this diatom under three silicon stress were captured to detect the phenotypic change. And then, Partial Least Square Regression (PLSR) models were constructed to predict the contents of polysaccharide, total lipid, protein and chlorophyll a (Chl-a) of *C. cryptica.* Moreover, the potential transformation links of these four products were established revealed by Intra-ramanome Correlation Analysis (IRCA).

## Materials and methods

### Microalgal species and culture conditions

The marine microalga (*C. cryptica*) was provided by the Lab of Applied Microalgae Biology of Ocean University of China (LAMB, Yushan, Qingdao, China). *C. cryptica* cells were cultured in F/2 medium [24, 25], the salinity of the culture solution was 30 ± 1. In addition, the culture system was put into a light chamber in which the temperature and intensity of illumination was controlled at 20 ± 1 °C and 80 μmol/m^2^/s (12 h light/12 h dark), respectively.

### The protocol for the testing

At first, *C. cryptica cells were* cultured in F/2 medium, the silicon content added according to the standard of F/2 medium. When the microalgal cells grew to the exponential growth period, which were centrifuged (3500 rpm, 10 min), and the sediment were inoculated into 3 L plastic bottles with 1 L different F/2 media including Si+ group (silicon-replete F/2 medium, control group) and Si− group (silicon starvation F/2 medium), respectively. For the Si−+ group, the microalgae cells were collected from the Si− group after 96 h of silicon starvation, and then re-cultured in the F/2 medium with the silicon-replete (That was, the 96 h in Si− group was the 0 h in Si−+ group). Three independent cultures for each timepoint in per group, and the initially cell number in three groups was adjusted to 9.8 × 10^5^ cells/mL. The sample of Si+ and Si− medium groups was collected at 0, 12, 24, 48, 72 and 96 h. The sample of S−+ medium group was collected at 0, 12, 24, 48, 72 and 96 h after 96 h silicon starvation in Si− group. The bottles were shaken manually three times a day during the experiment.

### Content of four intracellular target products measured by conventional approaches

#### The total lipid content

Total lipid was extracted according to the previous method [26, 27]. The *C. cryptica* culture were collected by centrifugation (3500 rpm, 10 min) and lyophilized overnight by an ALPHA 1–4 LD freeze dryer (Christ, Osterod, Germany). 50 mg of freeze-dried microalgal powder were put into a 100 mL centrifuge tube. 2.5 mL of chloroform, 5 mL of methanol and 2 mL of 50 mM K_2_HPO_4_ buffer (pH 7.4) were added into the centrifuge tube and shook for 2 h; After 2 h, 2.5 mL of chloroform and 2.5 mL of 50 mM K_2_HPO_4_ buffer (pH 7.4) was added into the above system again, mix uniformly and stood still. The liquid layered in the lower layer (chloroform layer) was transferred to a dry glass tube which the weight was taken as m_1_, and the glass tube with the liquid was put into a water bath at 60 °C until the liquid volatilizes completely, which the dried glass tube was weighed and was recorded as *m*_2_. The total lipid content (LC) was calculated as follows:

$$\mathrm{LC }(\mathrm{\mu g}/\mathrm{mg\,DW})=\frac{{m}_{2}-{m}_{1}}{50}$$

#### The polysaccharide content

The extraction of microalgal cell polysaccharide was based on the conventional method [28]. The content of polysaccharide was determined by phenol sulfuric acid colorimetry method [4]. Specifically, the polysaccharide was extracted and then hydrolyzed by the addition of 5% phenol and sulfuric acid in a boiling water bath. The optical density of the sample solution was determined by a UV-3310 spectrophotometer (Hitachi, Tokyo, Japan) at 490 nm. The total polysaccharide concentration was calculated based on a calibration curve using glucose as the standard. The glucose standard curve was *y* = 9.7445*x* − 0.0059 (*R*^2^ = 0.9974), which was established ahead. The total polysaccharide content (PC) was calculated as follows:

$$\mathrm{PC }(\mathrm{\mu g}/\mathrm{mg\,DW})=\frac{C*V*1000 }{m}$$

Here, *C* was the concentration of total polysaccharides (mg/mL), *V* was the final constant volume in the volumetric flask (mL) and *m* was the used dry weight of freeze-dried microalgal powder (mg).

#### The protein content

The content of protein was determined by Coomassie brilliant blue assay kit (Nanjing Jiancheng, China). In detail, 10 mL of *C. cryptica* culture was centrifuged (3500 rpm, 15 min) at room temperature. The supernatant was discarded and the harvested cell pellet was resuspended in 5 mL of double distilled water (dd-water) and was crushed by an ultrasonic cell crusher under 25% power for 20 min (Ningbo Scientz Biotechnology CO..LTD). The concentration of soluble protein in the supernatant was measured by the assay kit.

#### The pigment content

The pigment was extracted according to the method described in previous studies [30–32]. In detail, 10 mL of *C. cryptica* culture was centrifuged (3500 rpm, 15 min). The supernatant was discarded and the sediment were resuspended in 10 mL of 90% methanol, centrifuged after incubated in a 60 °C water bath for 15 min. The absorbance of the centrifuged supernatant at 665 and 652 nm wavelengths was measured with the UV-2000 spectrophotometer, respectively. Chl-a content were calculated by the formulas [32] as follows:

$${\text{Chl-a concentration }}({\upmu}{\text{g}}/{\text{mL}}) = 16.82{A_{665}} - 9.28{A_{652}}$$

$$\mathrm{Chl-a content}(\upmu \mathrm{g}/\mathrm{mg\,DW})=\frac{C*V}{1000*M}$$

Here, *C* was the Chl-a concentration (mg/L), *V* was volume of microalgal solution (mL) and *M* was the cell dry weight (g).

### Simultaneous quantification of polysaccharide, total lipid, protein and Chl-a at the single-cell level via SCRS

#### Acquisition of SCRS

SCRS were measured using a modified Horiba Lab Ram HR with an excitation wavelength of 532 nm [33]. In detail, 1.5 mL of microalgae culture was collected at the timepoint and centrifuged at 3500 rpm for 10 min. The supernatant was discarded, the sediment was washed with dd-H_2_O for three times, and was loaded into a capillary tube (50 mm length × 1 mm width × 0.1 mm height, Camlab, UK). The Raman spectra of 30 cells and four background sites in each of the three biological replicate cultures (i.e., 30 cells per biological replicate; three biological replicate cultures per timepoint; a total of 90 cells per timepoint) were randomly recorded. In general, an individual cell was trapped, photo-bleached and measured by a 532 nm laser with about 25 mW output power [21]. Raman spectrum between 393.8 and 3341.3 cm^−1^ was acquired and the acquisition time was 2 s.

#### PLSR model establishment for the quantification of four cellular products

The raw SCRS were pre-processed by background subtraction, baseline correction (a polynomial algorithm with a degree of seven) and normalization with home-made R scripts (RamEx). Information-abundant region of 449.499 ~ 3050.17 cm^−1^ were extracted and normalized by division based on its area. PLSR models were established to predict the contents of four target components of *C. cryptica*. For example, a PLSR model to predict the polysaccharide content was constructed based on the averaged SCRS and polysaccharide content measured by conventional phenol sulfuric acid colorimetry method among 10 samples collected at 0, 24, 72, 96 h in Si+ group, collected at 12, 48 and 96 h in Si− group, and collected at 12, 48 and 96 h in S−+ group. While the PLSR model for predicting the content of polysaccharide was validated by the rest 6 samples collected at 12, 48 h in Si+ group, collected at 24, 72 h in Si− group, and collected at 24, 72 h in S−+ group. Moreover, the polysaccharide content of individual microalgal cell was predicted by the SCRS based on the PLSR model. PLSR were performed with R (Version 4.0). Graphics were produced via the ggplot2 package.

### Statistical analysis

Three independent cultures of per group at each timepoint were used in conventional approaches, the data were carried out in triplicates (*n* = 3) reported as the mean ± deviation. Meanwhile, three independent cultures of per group at each timepoint were also used at the single cell level via SCRS, and 30 cells were sampled for each biological replication (90 cells per group at each timepoint were used to analysis). One-way ANOVAs and Tukey’s test was performed to evaluate the effect of silicon on polysaccharide, pigment, total lipid and protein of *C. cryptica*. The values were considered to be significant when *p* < 0.05 and extremely significant when *p* < 0.01. SPSS25.0 software was used for statistical analysis, and all figures were plotted by Origin9.0 software.

## Results

### Four intracellular target products change of *C. cryptica* was determined by traditional methods

**Fig. 1:**
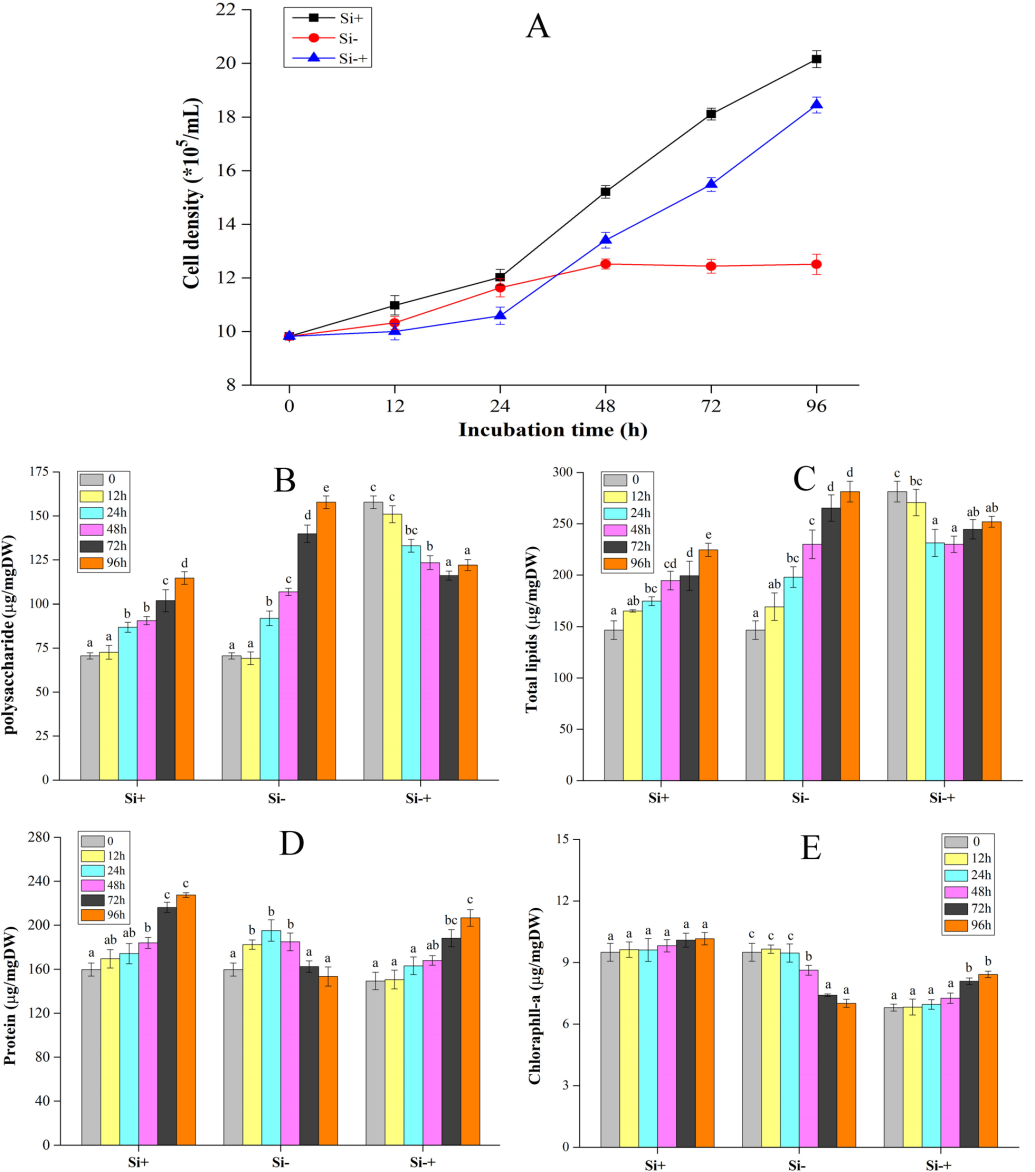
Cell density (**A**), polysaccharide (**B**), total lipid (**C**), protein (**D**) and Chl-a content (**E**) of *C. cryptica* in Si+ (silicon-replete F/2 medium, control group), Si− (silicon starvation F/2 medium) and Si−+ (silicon starvation F/2 medium followed by silicon-replete F/2 medium) media. Columns with different letters indicate statistically significant differences between treatments (*p* < 0.05, Tukey’s test)

To test whether the contents of polysaccharide, total lipid, protein and Chl-a can be simultaneously quantified at both the population and single-cell level via SCRS, the stress response process of *C. cryptica* under Si−, Si+ and Si−+ conditions was employed as a model (Fig. 1A). The contents of polysaccharide, total lipid, protein and Chl-a were measured via separately conventional approaches which followed the aforementioned “one procedure per target compound” paradigm (“Materials and methods”). Figure 1B shows that the content of polysaccharides in Si+ and Si− groups increased with the prolong of time. However, the content of polysaccharide in silicon starvation group increased faster than that in Si+ group. For example, the content of polysaccharides in Si+ group at 0, 12, 24, 48, 72 and 96 h was 71 ± 2, 73 ± 4, 87 ± 3, 91 ± 2, 102 ± 6 and 115 ± 4 μg/mg DW (Dry Weight), respectively. In addition, the content of polysaccharides in Si− group at the same timepoint was 71 ± 2, 69 ± 4, 92 ± 4, 107 ± 2, 140 ± 5 and 158 ± 4 μg/mg DW. Meanwhile, the polysaccharide content of Si−+ group decreased first and then increased.

Similar to polysaccharide, the content of total lipid in Si− group increased gradually with the time, and their values were higher than those in Si+ group within 96 h (Fig. 1C). For example, the total lipid content in Si− group at 0, 12, 24, 48, 72 and 96 h was 147 ± 9, 169 ± 13, 198 ± 10, 230 ± 13, 265 ± 13 and 281 ± 10 μg/mg DW, respectively, while the total lipid content in Si+ group at the same timepoint was 146 ± 9, 165 ± 1, 174 ± 4, 195 ± 9, 199 ± 14 and 225 ± 6 μg/mg DW, respectively. Similar to the change trend of polysaccharide content in S−+ group, the total lipid content of Si−+ group also decreased first and then increased, which the value decreased to the lowest value at 48 h (230 ± 8 μg/mg DW) and then began to increase.

Differ to the change trend of polysaccharide and total lipid in Si− and Si+ groups, the protein content in Si− group increased first and then decreased with time, for example, the protein content in Si− group decreased from 195 ± 10 μg/mg DW at 24 h to 153 ± 9 μg/mg DW at 96 h (Fig. 1D). The protein content of Si−+ group increased continuously within 96 h, which increased slowly in the first 48 h. However, the change trend of protein content in Si+ group was similar to that of polysaccharide and total lipid in Si+ group.

Chl-a content of Si+ group remained basically stable during the treatment cycle, which value ranged from 9.5 ± 0.4 to 10.2 ± 0.3 μg/mg DW (Fig. 1E). The content of Chl-a in Si−+ group showed similar to that in Si+ group. In contrast, the content of Chl-a in Si− group decreased continuously from 24 to 96 h, which the value decreased from 9.7 ± 0.2 μg/mg DW at 24 h to 7.0 ± 0.2 μg/mg DW at 96 h.

### Simultaneous quantification of four intracellular target products via SCRS

**Fig. 2:**
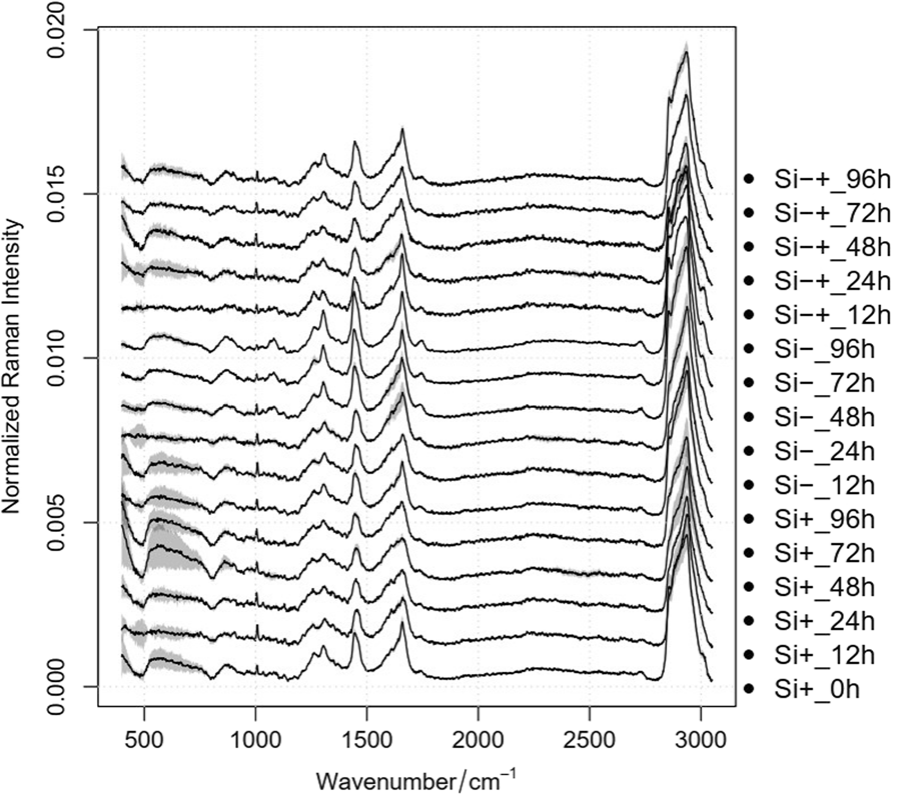
Temporal alteration of the averaged SCRS of *C. cryptica* with Si+, Si− and Si−+ treatments along the timepoint. Mean (solid lines) and standard error (shaded regions) from 90 individual cells are depicted

Cells of the above corresponding stress response process of *C. cryptica* under Si−, Si+ and Si−+ conditions were also collected at 0, 12, 24, 48, 72 and 96 h (i.e., 30 cells per biological replicate; three biological replicate cultures per timepoint; Fig. 2). The average Raman spectrum of *C. cryptica* changed regularly along with the timepoint and condition (Fig. 2). Among these, based on the Pearson correlation coefficient (*r*) between intensity of the Raman bands derived from SCRS and the corresponding quantitative trait, thirteen Raman bands were proposed as the main marker bands for the determination of these four intracellular target products (Table 1). Their intensity, when averaged at the set timepoint, exhibits positive correlation with the four target contents measured via conventional approaches.

**Table 1:** Main 13 reference Raman bands that are correlated with polysaccharide, total lipid, protein and Chl-a contents in *C. cryptica* during the process of three silicon treatment

Component	Raman brands (cm^−1^)	*r*	Assignment
Polysaccharide	1082	0.750	Carbohydrate C–O–H bending
	1262	0.226	Alkyl=C–H cis stretches
	2936	0.332	C–H_2_, C–H_3_ asymmetric and symmetric stretches
Lipid	1061	0.416	Alkyl C–C gauche stretches
	1303	0.406	Alkyl C–H_2_ twist
	1445	0.538	Alkyl C–H_2_ bend
	1658	0.568	Allyl C=C stretches
	2856	0.600	C–H_2_, C–H_3_ asymmetric and symmetric stretches
	2936	0.392	C–H_2_, C–H_3_ asymmetric and symmetric stretches
Protein	963	0.287	CH_2_, out-of-plane bending
	1005	0.442	Phenylalanine ring breath
	1610	0.362	C=O stretching of protein amide I; –NH_2_
Chlorophyl a	1005	0.685	Deformation of the methyl groups

Pearson Correlation coefficient (*r*) between averaged intensity of the Raman bands derived from SCRS and the corresponding quantitative trait shown

**Fig. 3:**
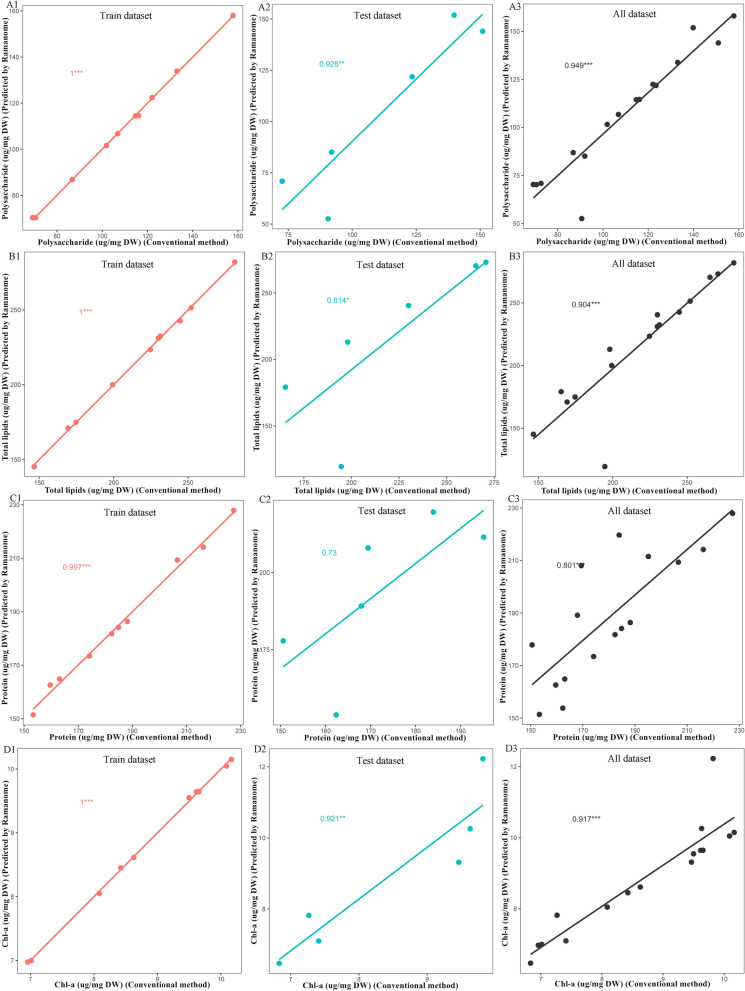
Quantification of polysaccharide (**A**), total lipid (**B**), protein (**C**) and pigment (**D**) contents in *C. cryptica* determined by SCRS method using PLSR models. The contents predicted by SCRS (*Y* axis) was plotted versus the corresponding value measured with conventional methods at the population level (*X* axis). 1 represents Train data set, 2 represents Test data set and 3 represents All data set (training data set plus test data set)

As the accuracy of a single Raman characteristic peak to label one phenotype is not high enough, we tried another strategy to predict cell phenotypes based on full spectrum modeling (i.e., PLSR model). For each timepoint, two of the triplicate cultures were used as training data set and the remaining one as test data set for model validation. For example, for polysaccharide content, the PLSR model was established using the averaged SCRS of 30 cells in one biological replicate and the corresponding polysaccharide content was also measured by the phenol sulfuric acid colorimetry method [29]. The *r* values of train data set, test data set and all data set were 1 (Fig. 3A1), 0.928 (Fig. 3A2) and 0.949 (Fig. 3A3), respectively. Similarly, the full spectrum-based PLSR model for total lipid, protein and Chl-a in single cell was also built and validated, achieving overall *r* of 0.904 (Fig. 3B), 0.801 (Fig. 3C) and 0.917 (Fig. 3D) (*p* < 0.01), respectively, indicating PLSR model could predict the content of four products with high accuracy. When averaged from that of the 90 cells as predicted by SCRS, each of the polysaccharide, total lipid, protein and Chl-a content, which was derived via the full spectrum, was highly consistent with those determined by conventional methods (Table 2), which verified again that SCRS was suitable for the determination of intracellular components of *C. cryptica*.

**Table 2:** Average contents of polysaccharide, total lipid, Protein and Chl-a of *C. cryptica* cells measured by Conventional method and SCRS

Groups	Content (μg/mg DW)	Conventional method						SCRS					
		0 h	12 h	24 h	48 h	72 h	96 h	0 h	12 h	24 h	48 h	72 h	96 h
Si+	Polysaccharide	71 ± 2	73 ± 4	87 ± 3	91 ± 2	102 ± 6	115 ± 4	70 ± 39	71 ± 39	87 ± 40	52 ± 35	101 ± 32	115 ± 42
	Total lipid	146 ± 9	165 ± 1	174 ± 4	195 ± 9	199 ± 14	225 ± 6	146 ± 58	179 ± 44	175 ± 52	119 ± 68	199 ± 51	224 ± 56
	Protein	160 ± 6	169 ± 8	174 ± 9	184 ± 5	216 ± 5	227 ± 2	162 ± 31	208 ± 30	174 ± 25	220 ± 35	214 ± 28	228 ± 23
	Chl-a	9.5 ± 0.4	9.6 ± 0.4	9.6 ± 0.6	9.8 ± 0.3	10.0 ± 0.3	10.2 ± 0.3	9.5 ± 1.8	10.3 ± 1.6	9.7 ± 2.1	12.3 ± 2.7	10.1 ± 1.7	10.1 ± 1.8
Si−	Polysaccharide	71 ± 2	69 ± 4	92 ± 4	107 ± 2	140 ± 5	158 ± 4	70 ± 39	70 ± 32	85 ± 48	107 ± 48	152 ± 38	158 ± 43
	Total lipid	146 ± 9	169 ± 13	198 ± 10	230 ± 13	265 ± 13	281 ± 10	146 ± 58	171 ± 53	213 ± 61	231 ± 63	271 ± 48	282 ± 55
	Protein	160 ± 6	182 ± 4	195 ± 10	185 ± 8	162 ± 5	153 ± 9	162 ± 31	182 ± 18	211 ± 25	184 ± 31	154 ± 20	151 ± 31
	Chl-a	9.5 ± 0.4	9.7 ± 0.2	9.5 ± 0.4	8.6 ± 0.2	7.4 ± 0.1	7.0 ± 0.2	9.5 ± 1.8	9.7 ± 1.6	9.3 ± 2.3	8.6 ± 2.2	7.1 ± 1.4	7.0 ± 1.9
Si−+	Polysaccharide	158 ± 4	151 ± 5	133 ± 4	123 ± 4	116 ± 3	122 ± 3	158 ± 43	144 ± 38	134 ± 47	122 ± 32	115 ± 28	122 ± 41
	Total lipid	281 ± 10	270 ± 13	231 ± 13	230 ± 8	245 ± 9	252 ± 5	282 ± 55	273 ± 45	232 ± 54	240 ± 44	242 ± 48	251 ± 57
	Protein	149 ± 8	151 ± 9	163 ± 8	168 ± 4	188 ± 8	207 ± 8	151 ± 31	178 ± 23	165 ± 23	189 ± 23	186 ± 20	209 ± 23
	Chl-a	6.8 ± 0.2	6.8 ± 0.4	7.0 ± 0.2	7.3 ± 0.3	8.1 ± 0.2	8.4 ± 0.2	7.0 ± 1.9	6.5 ± 1.7	7.0 ± 1.7	7.8 ± 1.7	8.1 ± 1.8	8.5 ± 1.5

Experimental data of Conventional method were reported as mean ± deviation (*n* = 3), and experimental data of SCRS were reported as mean ± deviation (*n* = 90)

**Fig. 4:**
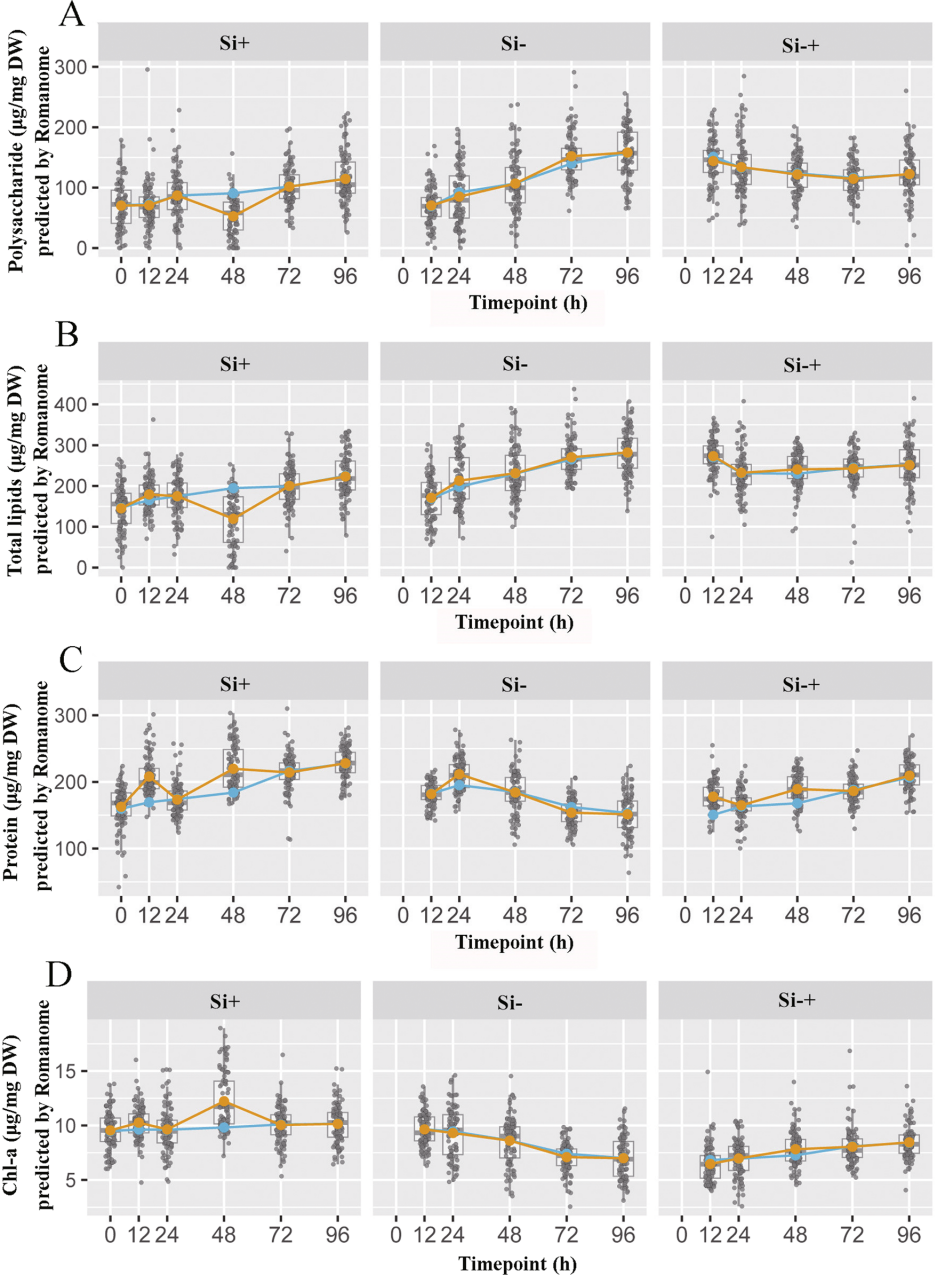
Contents of polysaccharide (**A**), total lipid (**B**), protein (**C**) and Chl-a (**D**) in individual *C. cryptica* cell acquired by SCRS. SCRS sampled from 90 cells and each data point represents one cell. The average contents of cells predicted by SCRS (yellow line) and measured by conventional methods are also shown (blue line)

**Fig. 5:**
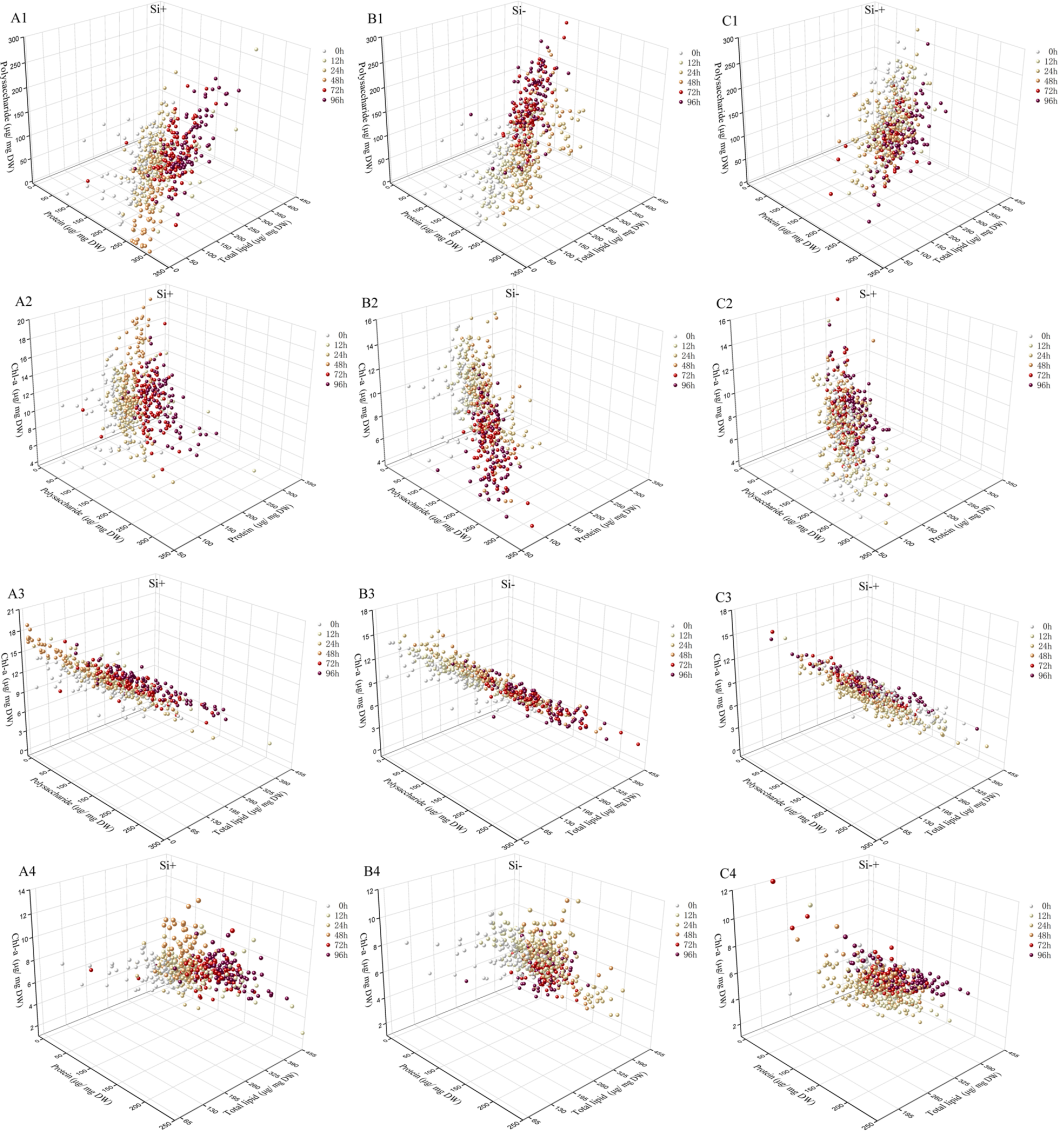
Temporal landscape of three biochemical components contents of individual *C. cryptica* cells in Si+ (**A**), Si− (**B**) and Si−+ (**C**) media. 1 represents polysaccharide–protein–total lipid, 2 represents Chl-a–polysaccharide–protein, 3 represents Chl-a–polysaccharide–total lipid and 4 represents Chl-a–protein–total lipid. Each data point represents one cell, with color indicating the timepoint

However, when the content of polysaccharide, total lipid, protein and Chl-a in 90 *C. cryptica* cells were simultaneously counted by SCRS to displayed the dynamic changes of four biochemical components in each cell, it could be seen from Fig. 4 that the content of four target cell components in different cells varies greatly. For example, in Si+ group, the content of polysaccharide at 72 h in one cell was 33 μg/mg DW, while the content in another cell was 198 μg/mg DW (Fig. 4A). Meanwhile, it could also be seen from Table 2 that the standard deviations of the data measured by the conventional method were far less than that measured via SCRS, which also revealed the difference in the component contents among cells. These results indicated that conventional methods require a large number of cells to reach the measured baseline while covering the differences between cells. Obviously, this was the advantage of SCRS method over conventional methods, which could capture the information of a single cell to screen specific phenotype cell. Moreover, simultaneous visualization of the polysaccharide, total lipid, protein and Chl-a content in each of the 540 *C. cryptica* cells sampled at 6 timepoints under three silicon media revealed the temporal landscape for microalgal energy storage compounds in the population at single-cell resolution (Fig. 5).

**Fig. 6:**
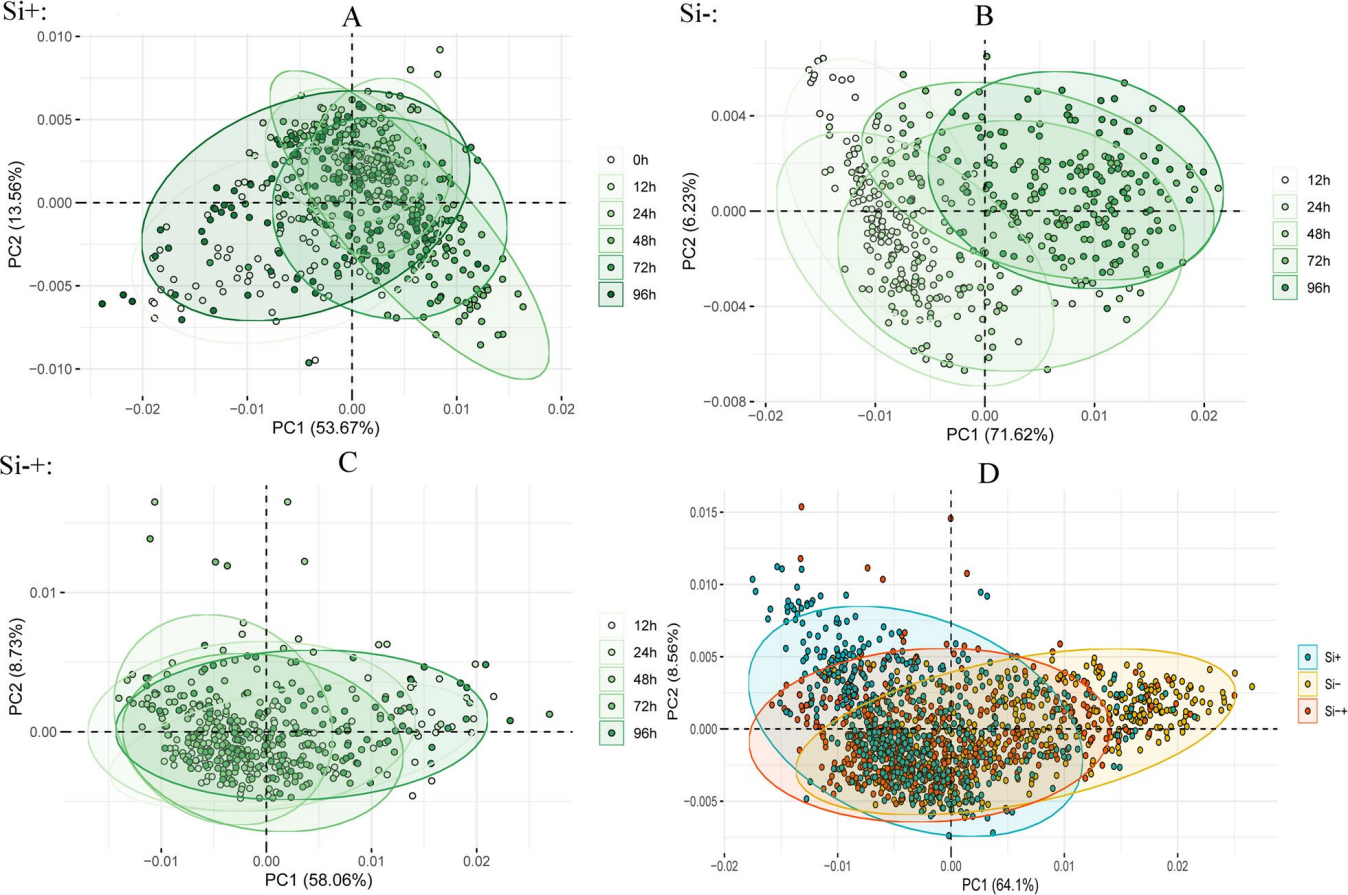
Cells of *C. cryptica* under Si+ (**A**), Si− (**B**) and Si−+ (**C**) treatments along the timepoint were displayed with Principal component analysis (PCA) based on their SCRS. **D** All cells under Si+, Si− and Si−+ treatments. Each data point represents one cell, with color indicating the timepoint or treatment

In addition, as SCRS can provide a sensitive biochemical ‘fingerprint’ of each cell, Principal component analysis (PCA) was used to visually demonstrate the cell-to-cell variability (Fig. 6). Clear differentiations were shown according to their growth time by the PCA scores plots, especially for that under Si− (Fig. 6B) and Si−+ treatment (Fig. 6C). In addition, cells under different silicon treatments can also be classified into different projective zones (Fig. 6D). These results indicated that the SCRS could be used to investigate the trend of cells in different states and different induction conditions [34].

### Instant interconversions among polysaccharide, total lipids, protein and Chl-a revealed by IRCA

**Fig. 7:**
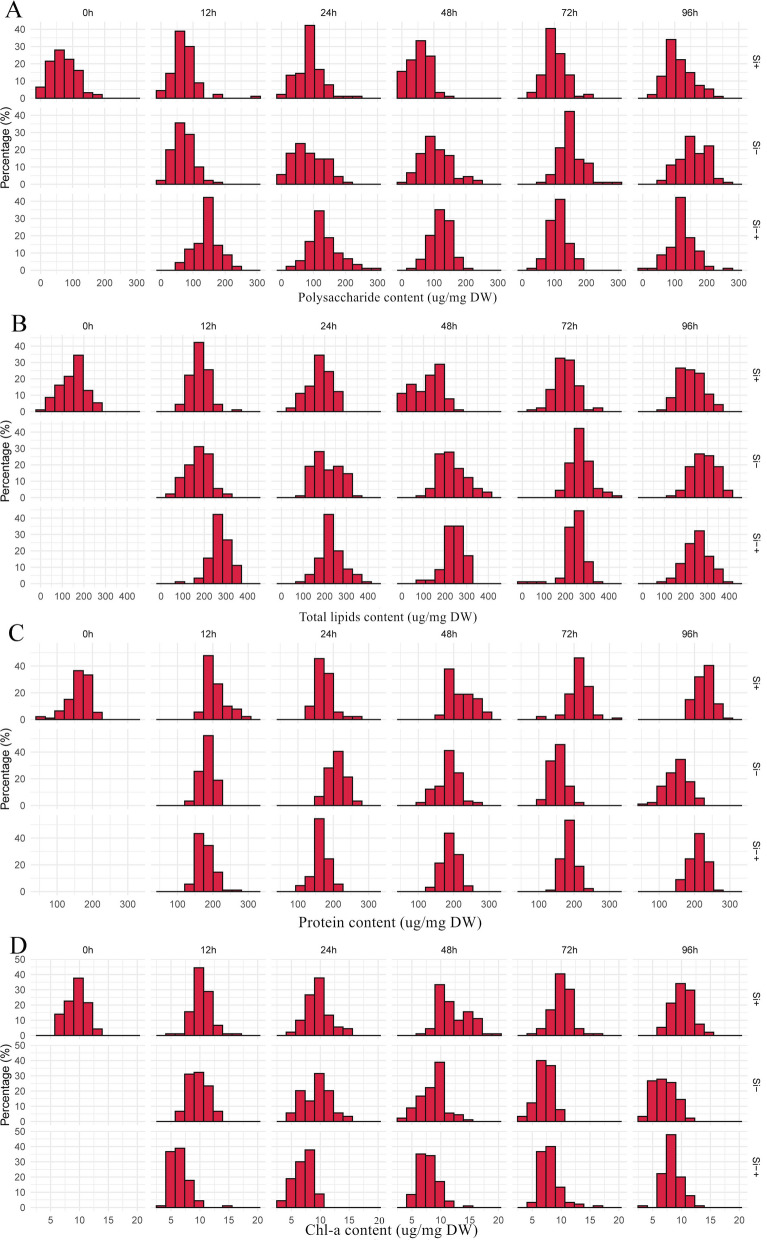
Phenotypic heterogeneity within the *C. cryptica* populations. Distribution of single-cell polysaccharide (**A**), total lipid (**B**), protein (**C**) and Chl-a (**D**) contents in the population at each of the 6 timepoints under Si+, Si− and Si−+ treatments. *X* axis is polysaccharide, total lipid, protein or Chl-a content in a cell (μg/mg DW) and *Y* axis is the frequency (%) of such cells

Interconversions among cellular components are the fundamental property for functioning a proper cellular system. To reveal these interconversions, time- or condition-series of samples is typically required. However, as the phenotypes of single-cell can be modeled, the measurement of their degree of among-cell heterogeneity in a given population come true [21]. For example, the phenotypic frequency distribution of the four cellular components indicated a high degree of heterogeneity in four intracellular components, which was prevalent in the *C. cryptica* population regardless of its state (Fig. 7). Heterogeneity within the total population indicates that caution should be taken in interpreting measurements of variables associated with cellular responses cellular responses [35]. Moreover, if we treat one cell as an independent sample, in a given population (certain condition and certain timepoint), the instant interconversion among polysaccharide, total lipids, protein and Chl-a could be reconstructed using the pairwise correlation among the cells of these four cellular phenotypes via IRCA [36].

**Fig. 8:**
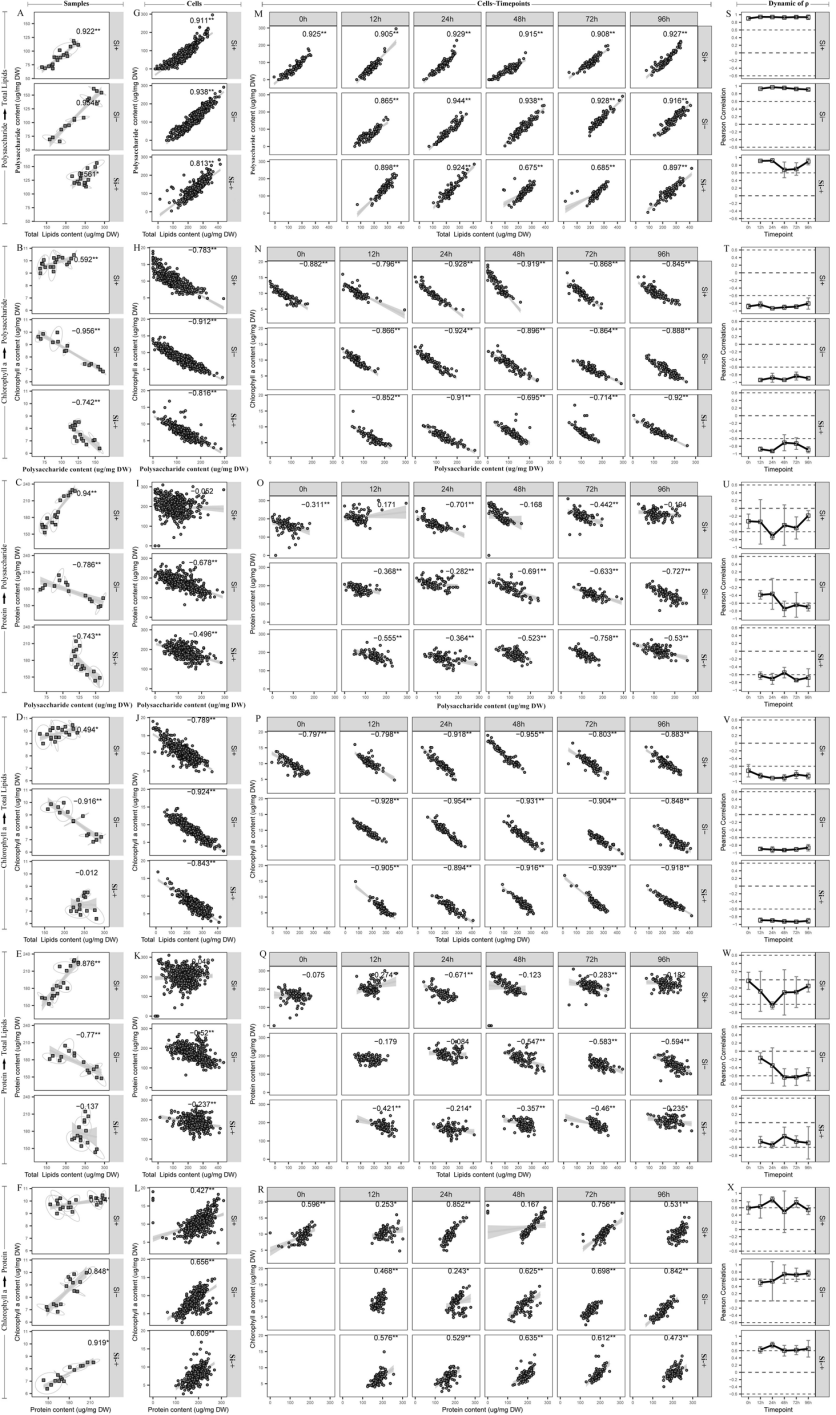
Interconversions among polysaccharide, total lipids, protein and Chl-a revealed by Intra-Ramanome Correlation Analysis of one isogenic cellular population. Pairwise correlation of polysaccharide, total lipid, protein and chlorophyll-a contents of *C. cryptica* at the population level and the single-cell level under Si+, Si− and Si−+ situations. Correlations of polysaccharide–total lipids (**A**), chlorophyll-a–polysaccharide (**B**), protein–polysaccharide (**C**), chlorophyll-a–total lipids (**D**), protein–total lipids (**E**) and chlorophyll-a–protein (**F**) contents at the population level were shown; Correlations of polysaccharide–total lipids (**G**), chlorophyll-a–polysaccharide (**H**), protein–polysaccharide (**I**), chlorophyll-a–total lipids (**J**), protein–total lipids (**K**) and chlorophyll-a–protein (**L**) contents at the single-cell level (**H**) were shown. Each dot in panels **A**–**F** represent one sample. Each dot in panels **G**–**L** represent one individual cell. **M** to **X** Correlation of any two biochemical components contents modeled by multiple pairs of singular Raman peaks among individual cells at each timepoint. Phenotypic correlation between total lipids (x) and polysaccharide (y) contents (**M** and **S**), between polysaccharide (x) and chlorophyll-a (y) contents (**N** and **T**), between polysaccharide (x) and protein (y) contents (**O** and **U**), between total lipids (x) and chlorophyll-a (y) contents (**P** and **V**), between total lipids (x) and protein (y) contents (**Q** and **W**), and between protein (x) and chlorophyll-a (y) contents (**R** and **X**) are also shown. ^*^Indicates statistical significance, *p* < 0.05 and ^**^Indicate statistical significance, *p* < 0.01. In the curves of temporal dynamics, *ρ* is the Pearson correlation coefficients of two phenotypes among single cells. There is strong correlation (*ρ* ≥ 0.6 or *ρ* ≤ − 0.6)

At the population level, significant correlation was observed in most of six phenotype pairs among the 18 populations under each silicon culture (Fig. 8A–F). For polysaccharide–total lipids, the correlation coefficient value at Si+, Si− and Si−+ group was 0.922, 0.954 and 0.561, respectively (Fig. 8A), which illustrated polysaccharides and total lipids were always synthesized simultaneously in these three silicon culture environments, and the degree of induction was Si− group > Si+ group > Si−+ group. For Chl-a–polysaccharide, the correlation coefficient value at Si+, Si− and Si−+ group was 0.592, − 0.956 and − 0.742, respectively (Fig. 8B), which indicated that Chl-a and polysaccharide synthesized simultaneously only at Si+ group, but Chl-a converted to polysaccharides at both Si− and Si−+ groups. Protein–polysaccharide, Chl-a–total lipids, protein–total lipids showed a similar result to the Chl-a–polysaccharide (Fig. 8C–E), these results indicated protein and polysaccharide, Chl-a and total lipids or protein and total lipids synthesized simultaneously at Si+ group while the conversions of protein to polysaccharide, Chl-a to total lipids or protein to total lipids at Si− and Si−+ groups. Chl-a–protein showed a similar result to that of polysaccharide–total lipids, that was, Chl-a and protein were also synthesized simultaneously in three silicon culture environments (Fig. 8F).

At the single-cell level, correlations for the 540 cells collectively reached a consistent conclusion to the population level (Fig. 8G–L). The correlation coefficient values of polysaccharide–total lipids and Chl-a–protein at Si+, Si− and Si−+ groups were strong positive correlation, which indicated polysaccharides and total lipids or Chl-a and protein were synthesized simultaneously in three silicon culture conditions, which were consistent with that at the population level. However, the correlation coefficient values of protein–polysaccharide, Chl-a–total lipids and protein–total lipids were also negative at Si− group and Si−+ group, that were also consistent with that at the population level. For example, the correlation coefficient value of Chl-a–polysaccharide and protein–polysaccharide in Si+ groups at single-cell level was − 0.783 (Fig. 8H) and − 0.052 (Fig. 8I), respectively, which were different from the positive correlation of 0.592 (Fig. 8B) or 0.94 (Fig. 8C) in Si+ group at the population level. These results indicated, at the single-cell level, the conversions of protein to polysaccharide, Chl-a to total lipids or protein to total lipids at Si− and Si−+ groups as well, thus, the results indicated the inter-phenotype correlation from all cells could recapitulate that among the populations [35]. The explanation of these observations might be that the correlations among cellular component are highly dependent on the sampling size. At the population level, Chl-a and polysaccharide or protein and polysaccharide in Si+ group was synthesized simultaneously, however, what happened at the single cell level was the conversion of Chl-a to polysaccharides or protein to polysaccharides in Si+ group, the result at single cell level could better reveal the instant interconversion process of intracellular substance than that at the population level, which indicated that single cell screening was very necessary.

**Fig. 9:**
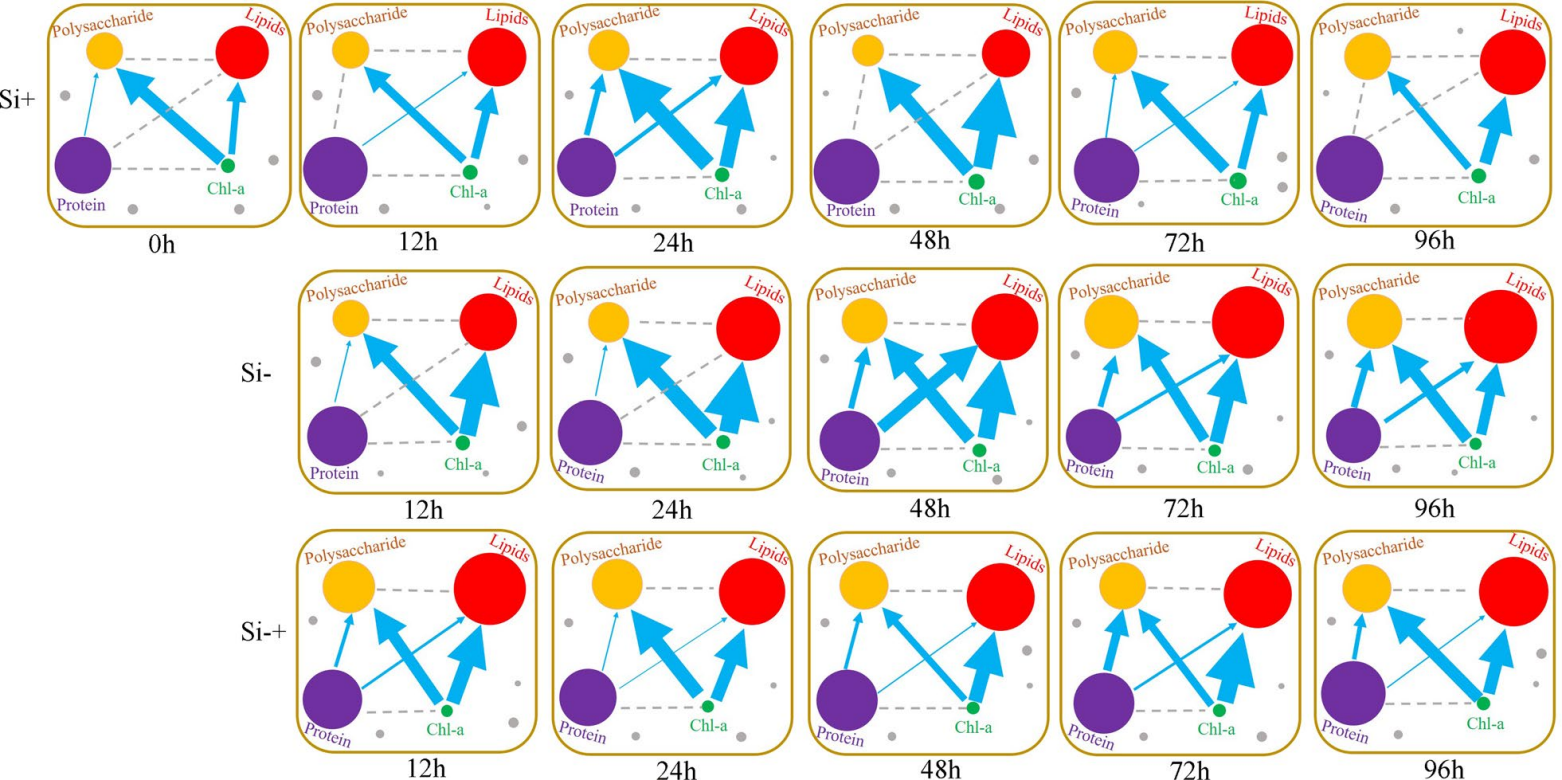
Choreography of interplay among major cellular components discovered by IRCA. The dot size represents the relatively product content, and the dot color represents different products (Yellow dots represent polysaccharides; red dots represent lipids; purple dots represent protein and green dots represent Chl-a). The blue arrow indicates that there is a negative correlation between products and the width of the arrow indicates the strength of the negative correlation. The arrow points to the potential flow direction of product conversion

Moreover, at three silicon treatment conditions, the correlation between inter-phenotype could be also detected at the set timepoint (Fig. 8M–R). For polysaccharide–total lipids, the positive correlation was strong at each of the timepoints in three silicon treatment cycles, for example, in Si− group, the correlation value of polysaccharide–total lipids at 12, 24, 48, 72 and 96 h was 0.864, 0.944, 0.938, 0.928 and 0.916, respectively (Fig. 8M, S), the correlation value of polysaccharide–total lipids at Si+ group and Si−+ group showed a similar trend to that in Si− group, which showed that the two components were all synthesized simultaneously at each timepoint in three silicon conditions. For Chl-a–polysaccharide, the situation was opposite to that of polysaccharide–total lipids, there was a negative correlation at each timepoint, especially in the Si− situation (Fig. 8N, T), which showed that the Chl-a–polysaccharide conversion took place at the whole phase of silicon treatments. For protein–polysaccharide, the result in Si+ group was different to that in Si− and Si−+ situations. The correlation value of protein–polysaccharide was 0.94 (significant positive correlation, Fig. 8C) at the population level, while a negative correlation at each timepoint were observed, which indicated that more detailed and instant cellular metabolism information can be revealed at the single cell level by IRCA. Meanwhile, a negative correlation of protein–polysaccharide in Si− situation were observed at each of the timepoints after 24 h and the temporal trend were strengthening (Fig. 8U). It is possible that the protein–polysaccharide conversion mostly took place at the late phase in Si− group. Moreover, the correlation was also different in three different silicon treatment situations. For example, the correlation value of protein–polysaccharide in Si+ medium at 96 h was − 0.194 that was no significant correlation, while the correlation value of protein- polysaccharide in Si− and Si−+ group at 96 h was − 0.727 and − 0.53, respectively (Fig. 8O), which indicated that the effect of silicon treatment on protein–polysaccharide conversion was Si− group > Si−+ group > Si+ group, that was, silicon starvation could promote the conversion of protein to polysaccharide. For Chl-a–total lipids, which was similar to Chl-a–polysaccharide, with strong negative correlation at each of the timepoints in three silicon treatments (Fig. 8Q, W), which indicated the conversion of Chl-a to total lipids also took place at the whole phase of silicon treatments. Protein–total lipids showed a similar trend to protein–polysaccharide, there was a strong positive correlation (0.876) of protein–total lipids at population level in Si+ situation (Fig. 8E), while a negative correlation at each timepoint was observed (Fig. 8Q). Therefore, the effect of silicon treatment on protein–total lipids conversion was also Si− group > Si−+ group > Si+ group. The possible reason for this phenomenon was the energy form originally used for protein synthesis has been transformed into the energy for polysaccharide and total lipid synthesis. For Chl-a–protein, there was a strong positive correlation at both population level and single cell level. This reason was like polysaccharides–total lipids, two components have the same change trend under silicon treatment stress. Altogether, a choreography of interplay among these major cellular components were displayed by summarizing the findings of IRCA (Fig. 9), which landscape-likely showed both the contents and links of mainly cellular components in corresponding to the cellular state and silicon treatment.

## Discussion

The diatom is an important part of the primary productivity of marine ecosystem because of its wide variety and large quantity [37]. Previous study has been reported that the contribution of diatom to marine primary productivity at global scale is estimated to be 30–40% [38], which played an irreplaceable role in maintaining carbon balance. Studies also have shown that silicon is a macronutrient required by diatoms to synthesize their silicified cell walls during their growth, as well as to understand the properties of key enzymes involved in flux of carbon into lipid also requires the participation of silicon [39–42] Obviously, the silicon plays a significant role in the growth and metabolism of the diatom. Thus, the biochemical components of *C. cryptica* under three silicon treatment conditions were first measured by conventional method. The results showed that the contents of polysaccharide and total lipids in Si− group increased rapidly, which were significantly higher than that in Si+ group, and the contents of protein and pigment in Si− group decreased after 24 h. By calculating the number of cells, it was found that the number of cells in the Si+ increased significantly, while the number of cells in Si− group stopped increasing after about 48 h (Fig. 1A), indicating the stagnation of cell growth and division. The growth condition of *C. cryptica* under silicon starvation obtained in this study was similar to that of *Thalassiosira pseudonana* in previous study described [40]. It could be seen from these results that silicon starvation could affect the division and growth of microalgae. Reasons for the changes might be the vigorous metabolism, rapid growth and strong fecundity of this diatom under the condition of sufficient nutrients, but the cells would not accumulate too much high energy storage substances, such as polysaccharides and lipids. When this diatom was cultured in the condition of silicon deficiency, the division ability was blocked. As this microalgal cells cannot divide normally, there is no good condition to continue to produce protein and pigment, leading to the decrease of their content. Meanwhile, these cells continue to fix carbon, so they have to do something with it. Usually, this goes to lipid and polysaccharide hyperaccumulation, as it is the least energy-expensive way to deal with the excess carbon coming in. Study also showed silicon deficiency may induce an increase in the rate of acetyl-CoA carboxylase, which was an important enzyme in the process of lipid synthesis [42]. Meanwhile, the sequencing results of nuclear genome and methylome of *C. cryptica* showed that the highly methylated repetitive sequences ensured the nuclear genome would not change significantly in the absence of silicon, but the annotation of pivotal glycolytic, lipid metabolism, and carbohydrate degradation processes revealed an expanded enzyme repertoire in *C. cryptica* in the case of silicon deficiency, which would allow for an increased metabolic capacity toward triacylglycerol production [43]. Therefore, the change of enzyme activity may be the main reason for the increase of total lipid content in *C. cryptica* exposed to silicon starvation stress. In addition, when *C. cryptica* were transferred to silicon medium after silicon starvation treatment, the content of total lipid and polysaccharide decreased gradually, which reached the minimum at 48 h and 72 h, respectively, and then increased, which corresponded to the growth of the microalgal cells. After silicon starvation treatment, the growth of *C. cryptica* stagnated, and a large number of high energy storage substances such as lipids and polysaccharides were accumulated in the cells. The growth of *C. cryptica* gradually recovered when they were transferred to the silicon medium again, and these energy storage substances were more used for cell division and growth, which would lead to the decrease of lipids and polysaccharides. Moreover, the number of cells increased greatly when the cells returned to the best state of growth, meanwhile, with the increased of cell density, intracellular energy storage substances also began to accumulate. Through this study, it was found that silicon starvation could promote the accumulation of lipids and polysaccharides in *C. cryptica*. This result in our study was similar to that in previous article, in which the researchers used flow cytometry imaging technology to reveal that silicon starvation would cause hyperaccumulation of triacylglycerol (TAG) in cell, and the majority of the cell volume was comprised of lipid droplets [35]. Therefore, silicon deprivation could be used to improve the production of valuable products, such as lipids in *C. cryptica*. Obviously, it is of great significance to screen microalgae strain with specific phenotypes, such as high lipid.

However, in microalgal cultures, most analyses of cellular processes are done on the entire population of cells and information gained from this is representative of the mean; however, it obscures the richness of cell-to-cell variation [35]. Meanwhile, the conventional methods also followed by tedious and time-consuming analytical procedures [21, 27, 29, 31, 32]. Thus, it is necessary to establish a simple and rapid method for determination of multicellular components in *C. cryptica*. SCRS is the superposition of molecular vibration modes of all components in the cell, which reflects the multi-dimensional information of the composition and content of chemicals in a specific cell [18, 19, 44]. It could characterize the composition and relative content of all metabolites in individual cell under specific condition [17, 45, 46]. Thus, the determination of multi-specific phenotypes of microalgae based on SCRS was practicable. Besides, the detection of SCRS was simple, noninvasive and rapid [19, 21], so the application of SCRS would save cumbersome detection procedures, greatly save human and material resources, and improve the screening efficiency of strains, which was in line with the rapid development of algae industry.

In recent years, SCRS has been used to determine the biochemical components of microalgae including *Cyclotella meneghiniana* [20], *Thalassiosira pseudonana* cell [16], and *Chlamydomonas reinhardtii* [21] etc. These results showed that SCRS can be used to detect the biochemical components of microalgae. However, this method has not been reported to analyze the biochemical components of *C. cryptica*. Therefore, SCRS was first attempt to determine these four biochemical components in this diatom. The results showed that the average change trend of polysaccharide, total lipid, protein and Chl-a contents of 90 cells under three silicon treatment conditions was consistent with the results of that measured by conventional methods, and the correlation coefficients of polysaccharide, total lipid, protein and pigment determined by SCRS and conventional method were about more than 0.9, indicating that SCRS was also suitable for the analysis of biochemical components of *C. cryptica*.

Moreover, in addition to the accuracy of measurement results, SCRS could allow detailed interrogation of individual cell and reveals cell-to-cell variation [18]. Through the analysis of four cell components by SCRS, we could also clearly and intuitively see that there were significant differences in the response of microalgae cells to the same environment. For example, the intracellular components of 90 cells at Si− group were significantly different, and even the maximum value in one cell was more than 10 times higher than the minimum value in another cell under the same silicon treatment conditions. Meanwhile, on the basis of Raman spectrum, IRCA could reconstruct a network of potential metabolite conversions among the four biochemical components using the pairwise correlation among the cells of the thousands of Raman peaks in SCRS [21]. The dynamic process of energy transformation between different cells could be seen more clearly, that was, the metabolite transformation reaction of cells in the instant state, while the transformation between substances observed at the population level was based on time- or condition-series of samples and not an instant cell response. Thus, SCRS could quickly reveal the transformation trend between substances through the association between multiple cells in one sample, so that we could more carefully understand the instant response state of cells under environmental stress, which was far more efficient than the population level. Through the result of IRCA, we could see that the content of polysaccharide and total lipid of *C. cryptica* at Si− treatment group increased followed by the decreased of protein and pigment, and the potential metabolic link intuitively showed the direction of these products metabolism with time at Si− condition (Fig. 9).

Briefly, the conventional methods revealed the average state at the population level, which were based on a large number of cell cultures to reach the baseline of the measurement, and SCRS could allow detailed interrogation of individual cell and reveals cell-to-cell variation. Consequently, SCRS was more suitable and rapidly for screening cells with specific phenotypes than traditional methods. A cell or a kind of microalgal cells with specific characteristic such as high polysaccharide or high lipid could be screen out through SCRS analysis, and then single-cell sequencing analysis, including transcriptome [47], proteome [48], metabolome [49], etc., which was more targeted for the analysis of cell molecular mechanism and could help us obtain more relevant accurate results.

## Conclusion

In this study, SCRS was first used to analyze the changes of biochemical components of *C. cryptica* cells under three silicon treatment conditions. First, the methods based on SCRS to simultaneously quantify the polysaccharide, total lipid, protein and pigment in single *C. cryptica* cell are established. In addition, the instant interconversion process of intracellular four components were constructed through IRCA, which is based on data set of one isogenic population and more precision and timeliness. Finally, total results indicated that silicon starvation could promote the carbon in *C. cryptica* cells to flow from protein and pigment metabolism to polysaccharide and lipid metabolism.

## Data Availability

The data that support the findings of this study are available from the corresponding author, Yun Li, upon reasonable request.
